# Immunomodulation and Disease Tolerance to *Staphylococcus aureus*

**DOI:** 10.3390/pathogens4040793

**Published:** 2015-11-13

**Authors:** Zhigang Li, Adam G. Peres, Andreea C. Damian, Joaquín Madrenas

**Affiliations:** Microbiome and Disease Tolerance Center, Department of Microbiology and Immunology, McGill University, Montréal, QC, H3A 2B4, Canada; E-Mails: zhigang.li@mail.mcgill.ca (Z.L.); adam.peres@mail.mcgill.ca (A.G.P.); andreea.damian@mail.mcgill.ca (A.C.D.)

**Keywords:** *Staphylococcus aureus*, immunomodulation, disease tolerance, microbiome, interleukin-10

## Abstract

The Gram-positive bacterium *Staphylococcus aureus* is one of the most frequent pathogens that causes severe morbidity and mortality throughout the world. *S. aureus* can infect skin and soft tissues or become invasive leading to diseases such as pneumonia, endocarditis, sepsis or toxic shock syndrome. In contrast, *S. aureus* is also a common commensal microbe and is often part of the human nasal microbiome without causing any apparent disease. In this review, we explore the immunomodulation and disease tolerance mechanisms that promote commensalism to *S. aureus*.

## 1. Introduction

*Staphylococcus aureus* is a Gram-positive bacterium of the *Micrococcae* family [1]. It has a thick cell wall composed of approximately 50% peptidoglycan (PGN) by weight [1]. *S. aureus* is commonly found as commensal bacteria located superficially on the human anterior nares, its primary reservoir [1,2]. From the nostrils, the bacteria can further spread to other sites such as the throat, skin, vagina, perineum, and the gastrointestinal tract [2].

Although it is a commensal, *S. aureus* is well equipped with a variety of virulence factors such as microcapsules, toxins, and drug resistance genes that contribute to pathology [1]. Infections with *S. aureus* occur with a seasonal trend, being highest in the summer and lowest in the winter [3]. *S. aureus* is the primary cause of local skin and soft tissue infections (SSTI) such as impetigo, dermatitis, and cellulitis [1,4]. Systemic infections caused by *S. aureus* include bacteremia, pneumonia, osteomyelitis, endocarditis, sepsis, and toxic shock syndrome (TSS) [4]. Invasive diseases by *S. aureus* have the highest annual death toll for any single infectious agent in the US, with close to 20% mortality [3,4]. One reason for this high death toll is the presence of methicillin-resistant *S. aureus* (MRSA). MRSA caused an estimated 80, 641 infections in US during 2011. Of these infections, 78% occurred in outpatients or within the community and carried a mortality rate of over 10% [5].

At any moment in time, 30%–50% of healthy adults are colonized with *S. aureus* and a fewer amount of those (around 20%) are persistently colonized [1,2]. It can be argued that everyone at some point is a carrier of *S. aureus* since serum immunoglobulin G (IgG) antibodies against staphylococcal antigens can be found in the entire population despite the lack of history of *S. aureus* infection [6]. *S. aureus* carriage rates differ based on ethnicity, gender, and age [7]. Currently, MRSA carriage rates remain much lower than *S. aureus* carriage but are gradually increasing [7]. In addition to humans, other natural reservoirs of *S. aureus* include cattle, sheep, and goat, yet the strain lineages appear to have evolved distinctively in each host [8]. *S. aureus* is reported to have a large economic impact in the dairy industry since it is the primary cause of mastitis [8]. Mice are not a natural reservoir of *S. aureus* and display a relative resistance to both the bacterium and its toxins, something to keep in mind when considering mouse models of disease [2,9].

We have termed *S. aureus* a pathobiont [10]. This classification refers to microbes that are regularly safe for their host but under certain conditions, other than immunosuppression, can become pathogenic. The switch between commensalism and pathogenicity is an important research topic because carriers have a greater risk of infection with their endogenous strain [2]. Over 90% of adult atopic dermatitis (AD) patients have either *S. aureus* colonization in their nares or skin [2], and 80% of *S. aureus* bacteremia cases in carriers were caused by their endogenous strain [11]. In fact, commensal isolates have been shown to contain most of the virulence factors and microbe-associated molecular patterns (MAMPs) that correlate with disease. Remarkably, carriers of *S. aureus* tend to have better outcomes to *S. aureus* bacteremia than non-carriers [2,11,12]. It is hypothesized that this difference in outcomes is due to varying degrees of immunomodulation or “priming” during *S. aureus* colonization [2].

## 2. Innate and Adaptive Immune Responses to *S. aureus*

### 2.1. Innate Immunity to S. aureus

The uppermost layer of the skin, the corneal layer, acts as a physical barrier to the microbiota, including *S.* *aureus.* In addition, chemical “elements” of defense such as anti-microbial peptides (e.g., human β-defensins 2 and 3) are secreted to the outer layer to control bacterial growth and breaching [4]. Below this protective cover, multiple cutaneous layers contain cells that will act as the first responders to *S. aureus*. Phagocytic cells and keratinocytes recognize MAMPs through their pathogen recognition receptors (PRRs) triggering a cutaneous immune response [13]. PRRs are a class of molecules that include cytosolic receptors (e.g., NOD-like receptors (NLRs), and RIG-I like receptors (RLRs)), secreted molecules (e.g., pentraxins and collectins) [14], and trans-membrane receptors (e.g., toll-like receptors (TLRs) and C-type lectin receptors (CLRs)) [13]. In the context of *S. aureus* recognition, NOD1/2 recognize muramyl dipeptides, which are PGN-derived fragments [15]. CLRs bind to microbial sugars in a calcium-dependent manner and further associate with other cell surface receptors to enhance phagocytosis [14]. TLR2 recognizes components of the bacterial cell wall such as teichoic acid, lipoteichoic acid (LTA), and PGN-embedded lipopeptides [15].

Since TLR2 has been reported as the dominant receptor for *S. aureus* and other Gram-positive bacteria [16,17,18,19,20], it will be the main focus of this review. TLR2 often dimerizes with either TLR1 or TLR6, and once engaged recruits adaptor proteins, TIRAP and MyD88, and the serine/threonine kinases IRAK-1 and 4 to initiate signaling [21]. Recruitment of these proteins leads to the activation of two pro-inflammatory pathways: NF-κB and mitogen-activated protein kinases (MAPKs) [21]. Ultimately, this translates into production of pro-inflammatory cytokines (e.g., IL-1β, IL-6, TNF-α, and IL-12p70) and chemokines (e.g., CXCL8, CCL2, CCL3, and CCL4), which are essential in combating infection by facilitating and enhancing phagocytosis and inflammation [21].

Plasticity in TLR2 signaling has been reported depending on the cell type expressing the receptor and the structure of the TLR2 ligands. Recently, our group has reported the existence of two sets of MAMPs that can trigger either predominantly pro-inflammatory or predominantly anti-inflammatory responses to TLR2 signaling [22]. TLR2 signaling on monocytes and macrophages will lead to a primarily anti-inflammatory response characterized by IL-10 production [15]. This response is not secondary to an alternative activation of these cells or M2 phenotype. In contrast, TLR2 signaling on dendritic cells (DCs) leads to a T_H_1/T_H_17 pro-inflammatory response through the production of IL-12 and IL-23 [15].

At the cellular level, neutrophils are the first cells to attempt the clearance of *S. aureus* [23]. They phagocytose the bacteria and use agents such as hypochloric acid (HOCl) and oxygen radicals to destroy the engulfed microbe. These activated neutrophils may also release DNA into the extracellular matrix to produce neutrophil extra traps (NETs) that control microbial spread, and enhance the cytotoxicity of antimicrobial agents while limiting immunopathology. Following the uptake of bacteria, neutrophils typically undergo accelerated apoptosis and are cleared by macrophages through efferocytosis. This process results in clearance of the microbe and recovery of the inflamed tissue to homeostasis [23]. However, in some circumstances, *S. aureus* pathogenicity is enhanced by a neutrophil-rich environment [23,24]. Some of these mechanisms will be touched upon later in this review.

### 2.2. Adaptive Immunity to S. aureus

When innate immune mechanisms are not sufficient to clear the bacterial infection, adaptive immune mechanisms are activated. An adaptive immune response to *S. aureus* requires a mixture of T_H_1, T_H_17, and humoral antibody responses. For example in SSTI both cellular and humoral responses (primary or secondary) are observed [25]. A T_H_1/T_H_17 response is associated with the production of IL-1 and IL-17A to promote abscess formation at the site of infection [4]. Abscessification is the hallmark of *S. aureus* infections and is required for the clearance of bacteria via phagocytosis and oxidative burst [4].

The Hyper-IgE Syndrome (HIES) highlights the importance of the T_H_17 responses in controlling *S. aureus* infections. In this syndrome, a mutation in the *stat3* gene results in lower levels of retinoid-related orphan receptor (ROR) γt [26], the master regulator of T_H_17 lineage differentiation. Thus, these patients show defective IL-17A production and increased susceptibility to *S. aureus* infections [26]. HIES is manifested as a T_H_2 phenotype concomitant with the absence of T_H_17 cells [26] but it is not clear whether this clinical presentation is because of the lack of RORγt, IL-17A, IL-17A receptor (IL-17RA), or STAT3. It is known that IL-17RA deficiency leads to susceptibility to mucocutaneous *Candidiasis* but not to *S. aureus* [27]. In addition, a more recent publication noted that RORγt deficiency in itself results in susceptibility to *Candida* and *Mycobacterium* infections but not to increased susceptibility to *S. aureus* [28].

A T_H_1 response during *S. aureus* infections is commonly associated with TSS where staphylococcal superantigens (SAgs) activate roughly 20% of T cells leading to their massive proliferation and production of cytokines [29]. This activation occurs by the binding of the SAg to selective variable regions of the T cell receptor β-chain (TCR-Vβ) and to some MHC class II molecules on antigen-presenting cells (APCs) [29]. SAg expression is not exclusive to isolates from TSS-associated strains. In fact, the staphylococcal enterotoxin gene cluster (egc) containing 6 SAgs is commonly found in commensal isolates [29,30]. Another example is the staphylococcal enterotoxin A (SEA), which is well known for inducing a strong pro-inflammatory/T_H_1 response, and can be found in pathogenic as well as commensal isolates. However, this is not unique to SEA because all SAgs have been found to share similar superantigenicity when levels of pro-inflammatory/T_H_1 cytokines were measured *in vitro* [29].

In addition to T cell responses, humoral responses have been found to play a role in the outcome of *S. aureus* infections. In the serum of patients with *S. aureus* bacteremia, IgG antibodies targeting eight conserved extracellular proteins can be found. Of these targets, seven are coded by the bacterial core genome [6]. The proteins targeted by these antibodies include phospholipase and immunodominant staphylococcal protein A (IsaA) [6]. These antibodies may have prognostic value since higher affinity antibodies are associated to better outcomes for sepsis patients [6].

The role of immunological memory to *S. aureus* in humans is still unclear [2,31,32]. There is currently no clinical evidence to claim protective immune memory to *S. aureus*. This observation is in line with challenges we have in developing a vaccine against this microbe. In mice, recent studies suggest that IL-17A may be important for the development of a memory state because a high IL-17A-to-IFNγ ratio in mice has been linked to protective memory to SSTI [25]. This IL-17A-to-IFNγ ratio seems to be determined by the genetic background of the host [25]. This evidence would suggest that the development of immunological memory to *S. aureus* may reflect a balance between nominal antigen-induced T_H_17 responses and SAg-induced T_H_1 responses. If so, the former response would conduce to protection upon re-exposure, whereas the later would not. However, this may not be applicable to the same extent in humans because patients with a defect in RORγt or IL-17 receptor signaling, which lead to impaired T_H_17 responses, show no increased susceptibility to repeated *S. aureus* infections [27,28].

## 3. Cellular Basis of Immunomodulation by *S. aureus*

The capacity of a microbe to induce anti-inflammatory responses and to restrain host pro-inflammatory responses to that microbe is referred to as immunomodulation. In addition to triggering host innate and adaptive immunity, *S. aureus* has a plethora of sophisticated mechanisms that allow it to induce immunomodulation that promote disease tolerance and establish commensalism. The precise molecular and cellular basis of immunomodulation is starting to emerge.

Like other microbes, recognition of *S. aureus* relies on MAMPs binding to PRRs on host cells. These cells include epithelial cells, macrophages, DCs and neutrophils [10]. PGN from Gram-positive bacteria, which contains teichoic acid, LTA and lipoprotein, is the main source of MAMPs. TLR2 has emerged as a dominant receptor for some of these molecules [4]. The qualitative and quantitative representation of TLR2, 1, 6 and 10, as well as the accessory molecules CD14 and CD36 on these cells, will likely determine the spectrum of their response [33,34,35]. Recognition of MAMPs by PRRs leads to a strong release of pro-inflammatory cytokines and chemokines, as well as the anti-inflammatory cytokine IL-10 [36].

Although most *S. aureus* isolates express at least one SAg [37,38,39], the incidence of TSS in *S. aureus* carriers is low [40,41]. This paradox may be explained by the fact that the staphylococcal cell wall down-regulates SAg-induced T cell activation. *S. aureus* PGN-embedded TLR2 ligands induce IL-10 production and apoptosis of APCs, down-regulating SAg-induced T cell activation and preventing TSS [42]. Consistent with this mechanism, it has been reported that staphylococcal LTA is able to inhibit injury-induced skin inflammation through a TLR2-dependent mechanism [43].

In addition to cell wall components, secreted staphylococcal toxins can mediate immunomodulation in SSTI. In a murine model of dermonecrosis, infection with an α-toxin-deficient *S. aureus* strain resulted in enhanced innate and adaptive immune responses, compared with infection with wild-type strain, as illustrated by increased neutrophil infiltration, influx of innate IL-17^+^ γδ T cells, recruitment of T_H_1 and T_H_17 cells, and enhanced pro-inflammatory cytokine and chemokine production. These effects were correlated with reduced lesion size. The enhancement of the response to *S. aureus* was also observed with the administration of an α-toxin neutralizing antibody prior to the staphylococcal infection [44]. Altogether, these data suggest a role of α-toxin in immunomodulation.

Emerging evidence suggests that IL-10 is an important mediator of immunomodulation by *S. aureus*. IL-10 inhibits T_H_1 and T_H_17 responses to *S. aureus* [15]. In humans, IL-10 can also inhibit T_H_2 responses [45]. In addition to the effect on pro-inflammatory cytokines, IL-10 down-regulates production of some chemokines such as CCL3, CCL4, CXCL8, and CXCL10 [46], resulting in prevention of immune cell recruitment to inflammatory sites. Furthermore, IL-10 down-regulates expression of MHC class II [47] and co-stimulatory molecules on APCs [48,49], leading to decreased T cell activation.

Quantitatively, monocytes and macrophages are the main source of IL-10 in response to *S. aureus*. The production of IL-10 by monocytes and macrophages is between 4 and 20 times higher than that by DCs [15]. In contrast, when DCs are the predominant APC, *S. aureus* triggers a robust T_H_1/T_H_17 response [15]. This difference suggests that the capacity of immunomodulation by *S. aureus* is dependent on the type of APC. Consequently, one would expect different outcomes of *S. aureus* detection in different tissues based on different cellular composition. For example, in the nose, macrophages are the primary APCs, whereas in the skin Langerhans cells and other DCs are more abundant. This might explain why *S. aureus* usually acts as a commensal in the nose *(i.e.,* nasal carrier) and a pathogen in the skin (e.g., AD).

*S. aureus* can also induce immunomodulation through the regulation of monocyte/macrophage function and differentiation. In a mouse model of catheter-associated biofilm infection, *S. aureus* attenuated phagocytosis by macrophages, and polarized these cells towards an alternatively activated M2 phenotype, altogether down-regulating host inflammatory responses [50]. Targeting macrophage activation with administration of the C5a receptor agonist EP67 or with pro-inflammatory M1 macrophages reduced the staphylococcal bacterial burden [51]. In line with these findings, macrophages from chronic rhinosinusitis patients with nasal polyps show reduced capacity of *S. aureus* phagocytosis and are polarized to a M2 phenotype compared to macrophages from chronic rhinosinusitis patients without nasal polyps. This correlates with more staphylococcal colonization in the former patients. These findings suggest that alternative activation of macrophages contributes to *S. aureus* chronic infection and colonization [52]. The mechanism by which *S. aureus* skews macrophage differentiation has been linked to Akt1 signaling and subsequent regulation of the miR-155/SOCS1 axis [53].

In addition to monocytes and macrophages, T cells can produce IL-10 upon *S. aureus* infection. It is unlikely that *S. aureus* directly interacts with T cells to induce IL-10 production, since human resting T cells do not express TLR2 [54]. However, *S. aureus* can imprint T cells to produce IL-10. For example, it has been reported that *S. aureus* primes naïve T cells to differentiate into IL-10-producing T_H_17 cells [55]. In another *in vitro* polarization study, *S. aureus* imprinted neonatal cord blood CD4^+^ T cells to differentiate into FOXP3^+^CD25^+^CD127^l^°^w^ Treg cells through a PD-1/PD-L1 dependent mechanism [56].

Induction of IL-10 production by *S. aureus* can also facilitate immune evasion, which under certain circumstances is a form of immunomodulation. IL-10 inhibits pro-inflammatory immune responses and establishes an anti-inflammatory environment [46,57]. Under these conditions, there is decreased immune cell recruitment to the infection site, which contributes to reduced pathogen elimination and ultimately may lead to chronic infection.

Recent evidence has suggested the involvement of IL-10-independent mechanisms in *S. aureus*-induced immunomodulation. *S. aureus* or its PGN are able to down-regulate staphylococcal SAg-induced expression of chemokines such as CXCL10, and prevent T cell recruitment. Such a regulation is not due to IL-10 production, but rather to *S. aureus*-triggered activation of MAPKs p38 and ERK, and inhibition of STAT1 signaling (Li *et al.*, manuscript submitted).

Recently, a study on cutaneous infection by *S. aureus* has demonstrated a role of Gr1^+^CD11b^+^ myeloid-derived suppressor cells (MDSCs) in immunomodulation [16]. Signaling through TLR2/6 but not TLR2/1 in skin resident cells triggers production of IL-6, which induces the accumulation of MDSCs. MDSCs are recruited to skin and suppress T cell-mediated recall responses. Bacterial lipoproteins are essential for this immune suppression. A chronic *S. aureus* infection model has corroborated the important role of MDSC in immunosuppression [58]. In this study, a robust IL-10 and TGF-β response was observed, but it was not essential for the immunosuppressive effect. In addition, Tregs did not play a significant role in either infection model.

## 4. Molecular Mechanisms of Immunomodulation by *S. aureus*

In addition to the pro-inflammatory properties, the cell wall of *S. aureus* can trigger an anti-inflammatory response. Emerging evidence indicates that TLR2 ligands embedded in staphylococcal cell wall are involved in *S. aureus*-induced immunomodulation. LTA is one of these ligands that down-regulates host innate and adaptive immune responses [43,59]. However, LTA is unlikely to be the only anti-inflammatory molecule, since PGN induces more IL-10 than LTA alone [15]. Furthermore, we detected higher induction of IL-10 by heat-killed *S. aureus* than commercially prepared staphylococcal PGN, which implies that ligands other than PGN-derived molecules are responsible for the immunomodulation. The exact immune regulatory molecules still need to be identified. In addition to cell wall components, the staphylococcal secreted protein Map (MHC class II analog protein) can act as an immunomodulator that interferes with T cell-mediated responses [60], although the molecular mechanism is yet to be investigated.

Recent studies have demonstrated that the pro-inflammatory properties and immunomodulatory properties of *S. aureus* can be uncoupled. Nasal *S. aureus* isolates have a different IL-10-inducing capacity, which does not correlate with the TNFα-inducing capacity [22]. The composition of the *S. aureus* cell wall may induce the formation of different TLR2 signaling complexes. The balance between these receptor complexes may determine the difference in anti-inflammatory capacity. For example, TLR2/6 dimers, rather than TLR2/1 dimers, have been often linked to immunomodulation [42]. Accessory molecules CD14 and CD36 are not required for IL-10 production, whereas CD14 is required for the pro-inflammatory response [15]. Phagocytosis and phagosomal processing of *S. aureus* are required for the pro- but not the anti-inflammatory response [22]. In addition, different intracellular signaling pathways also account for different immune responses to *S. aureus*. The PI3K/Akt/mTOR and extracellular signal-regulated kinase (ERK) pathways are responsible for the immunomodulation, while the MAPK p38 pathway is essential for pro-inflammatory response [22]. The MyD88 adaptor-like molecule TIRAP, but not MyD88, has been linked with TLR2/6-induced PI3K activation [61].

Signaling from TLRs trigger activation and nuclear translocation of several transcription factors that drive IL-10 expression. NFκB [62], C/EBPβ [63,64], ATF1 [65], IRF1 [66], STAT3 [66], and the transcription factors specific protein 1 (SP1) [67] and SP3 [68] complex at the IL-10 promoter to initiate IL-10 transcription in monocytes and macrophages.

**Figure 1:**
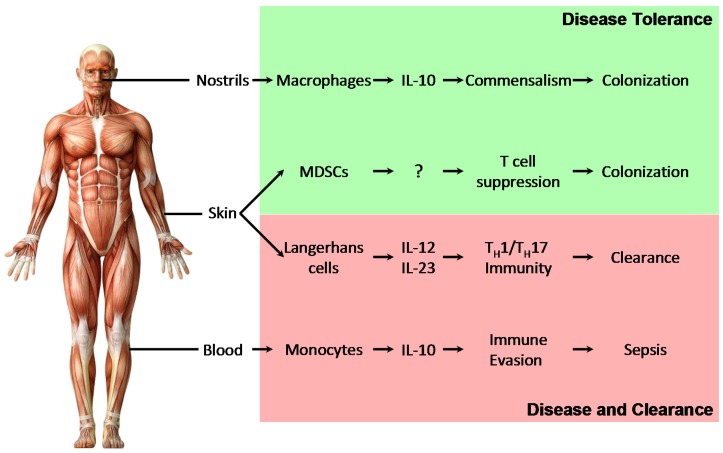
The outcome of *S. aureus* detection depends on the site of infection. In nostrils, macrophages, the primary antigen-presenting cells (APCs), produce high levels of IL-10 upon *S. aureus* stimulation, which promote commensalism and disease tolerance to *S. aureus*, and ultimately staphylococcal carriage. Upon cutaneous infection by *S. aureus*, myeloid-derived suppressor cells (MDSCs) are recruited to the skin and regulate T cell-mediated recall responses, which may facilitate disease tolerance to the microbe. In addition, Langerhans cells are the main APCs in skin, which activate a robust T_H_1/T_H_17 response, resulting in bacterial clearance. In blood, the primary APCs are monocytes, which produce abundant IL-10 upon staphylococcal infection. This facilitates immune evasion and may lead to sepsis. The human body illustration is from wiseGEEk (http://www.wisegeek.org/how-many-muscles-are-there-in-the-human-body.htm#anatomy-of-human-muscle). DC: dendritic cell; MDSC: myeloid-derived suppressor cell; IL-10: interleukin-10.

The properties of immunomodulation and commensalism by *S. aureus* might be determined by several factors, such as the site of infection, strain of *S. aureus*, host genetics, host diet and environment. The nostril is a common commensal colonization site of *S. aureus*, while skin colonization by this microbe is usually associated with disease, such as AD (Figure 1). In blood, infection by *S. aureus* can lead to severe sepsis. Capacity of immunomodulation by *S. aureus* varies among community isolates, which is uncoupled with its inflammatory capacity [22]. Higher IL-10-inducing isolates may have better colonization capacity, due to the tolerogenic environment generated by IL-10. A study on single nucleotide polymorphisms showed that human genetics play an important role in determining persistent nasal *S. aureus* carriage [69]. Although genotypes of C-reactive protein (CRP) and IL-4 are suggested to be the determinants, the detailed mechanism is still unknown. Diet and living conditions also affect *S. aureus*-host interaction. For example, the addition of vitamin D to a bovine mammary epithelial cell culture enhances the IL-10 response to *S. aureus* [70].

In addition to IL-10, TGF-β can play an anti-inflammatory role in microbe-induced immunomodulation [71]. For example, *Streptococcus pneumoniae* induces TGF- β expression and drives Treg infiltration, which is essential for pneumococci-induced immunomodulation and chronic carriage [72]. *S. aureus* can also induce production of TGF- β in several infection models [58,73]. However, the expression of TGF- β did not contribute to the immunosuppressive effect of *S. aureus* in this study [58].

## 5. Mechanisms of Disease Tolerance to *S. aureus*

During infection, *S. aureus* causes extensive tissue damage to its host either directly by its virulence factors (e.g., α-toxin), or through hyper-activation of the host immune system (e.g., immunopathology induced by staphylococcal SAg). The mechanisms of *S. aureus*-induced damage are balanced by “disease tolerance” mechanisms that prevent unnecessary tissue destruction caused by the microbe or the host [74]. Disease tolerance improves host fitness by limiting pathology and reducing mortality without directly affecting microbial burden [75]. These mechanisms are different from disease resistance mechanisms that aim at clearing the microbe. Disease tolerance has been reported in a variety of infection models [76,77,78]. Whether these mechanisms are involved in the establishment of commensalism is not yet known, but it is likely given that tissue integrity is a feature of commensalism. We hypothesize that disease tolerance is not only essential to limit *S. aureus*-induced tissue damage and pathology during infection but also for *S. aureus* to establish commensalism.

The importance of disease tolerance in limiting *S. aureus* pathogenesis was recently highlighted by the observation that autophagy prevents tissue damage caused by α-toxin [79]. In this study, the authors found that autophagy-deficient mice (*i.e*., Atg16L1^HM^ mice) were susceptible to *S. aureus* pneumonia and sepsis independently of bacterial burden. They established that susceptibility was conferred by production of the *S. aureus* α-toxin, and that autophagy down-regulated the expression of the α-toxin receptor ADAM10 in epithelial cells, thereby limiting damage during *S. aureus* infection. Interestingly, anthracyclines, a family of DNA damaging chemotherapeutic agents, also induced autophagy in the lung and protected mice from cecal-ligation and puncture sepsis [78], suggesting autophagy in the lung is an important disease tolerance mechanism to sepsis and, perhaps, other systemic *S. aureus* infections. Whether a similar effect is seen in the upper respiratory tract during *S. aureus* commensalism is currently unknown. However, given the importance of the integrity of the nasal epithelial layer during commensalism, it is reasonable to speculate that autophagy in the nose would confer disease tolerance to *S. aureus* as well. Indeed, autophagy-deficiencies have been identified in nasal polyps [80], and staphylococcal exotoxins have been found to dysregulate epithelium integrity [81,82]. Moreover, α-toxin expression in the nose is low [83,84], perhaps as a deliberate effort by *S. aureus* to limit pathology. It is, therefore, likely that autophagy mediates disease tolerance in the nose to promote *S. aureus* commensalism.

The hyper-active immune response during staphylococcal systemic complications causes severe tissue damage through immunopathology. In particular, the cytokine storm resulting from massive T cell activation by SAgs leads to multi-organ failure, a hallmark of staphylococcal TSS [85,86]. Any reduction of such an exacerbated immune response would likely slow down this progression and provide the host with additional time to clear the infection without the devastating immunopathology. We have previously shown that the IL-10 response induced by the staphylococcal cell wall is highly effective in suppressing SAg-induced T cell activation, and prolonging survival in a mouse model of staphylococcal TSS [42]. This mode of action likely has little effect on bacterial burden, but rather limits immunopathology by the over-active immune response. In support of this, staphylococcal TSS rarely develops (less than 5% of the time) during staphylococcal bacteremia [87,88], most likely because of elevated IL-10 levels [57] that protect against extensive tissue damage.

The breakdown of disease tolerance during staphylococcal TSS may explain the shortage of current treatments. The limited success of many of these treatments may be due to the focus on eliminating the microbe, and ignoring the extensive tissue damage that is occurring. Implementing treatment strategies that combine tissue damage control and antibiotics that inhibit toxin production (e.g., clindamycin [89]) may provide the most effective approach to combat TSS and other staphylococcal systemic infections.

Similar to staphylococcal TSS, immunomodulation through IL-10 may play a disease tolerance role during *S. aureus* commensalism. IL-10 is an essential anti-inflammatory molecule that mediates tolerance to the gut microbiota, and mutations and/or deficiencies in IL-10 cause severe enterocolitis in mice [90] and humans [91]. We predict a similar effect in the nose whereby IL-10 promotes *S. aureus* commensalism by limiting the inflammatory response to *S. aureus* and tissue damage caused by its toxins. For example, IL-10 would protect against nasal epithelium dysregulation caused by staphylococcal SAgs [92]. Indeed, nasal *S. aureus* isolates do carry SAgs as frequently as clinical isolates [39], but are not causing disease. Moreover, unlike *Streptococcus pyogenes* [93], staphylococcal SAgs are not required for *S. aureus* colonization in an HLA-DR4 transgenic mouse [22], perhaps due to the more robust IL-10 response it induces [42]. Higher IL-10 levels in the nasal epithelium are also associated with better tolerance to house dust mite allergens [94,95]. Unfortunately, studies on the function of IL-10 in the nose as it pertains to commensals are lacking. Nonetheless, the role of IL-10 in regulating *S. aureus* commensalism is an intriguing possibility that must be explored further. Additional disease tolerance mechanisms by particular receptors (e.g., aryl hydrocarbon receptor [96]) reported for other inflammatory conditions remain to be tested in *S. aureus* infections.

## 6. Mechanisms of Immune Evasion by *S. aureus*

Another important aspect of *S. aureus* pathobiosis is the ability of this microbe to evade the host immune response. Immune evasion can be broadly classified into two main types: evasion by avoiding host detection, or evasion by modulating the host immune response. Here, we will only briefly outline the mechanisms of *S. aureus* immune evasion as they have been recently reviewed extensively elsewhere [97,98].

To avoid detection, *S. aureus* produces a plethora of virulence factors to avert the essential components of the host immune response involved in the elimination of *S. aureus*. These include the complement system, leukocyte recruitment mechanisms, and phagocyte function. For example, *S. aureus* has developed multiple, parallel strategies to subvert opsonization and chemotaxis by the complement system. Some examples are the staphylococcal C3 convertase assembly inhibitor [99], fibrinogen-binding proteins that prevent C3b deposition on the staphylococcal cell wall [100] and C3 conversion to C3b [101], and proteases that cleave C3b into an inactive form [102]. In addition to inhibiting C3b, *S. aureus* also inhibits the anaphylatoxins C3a and C5a through proteolysis [102,103], or through receptor antagonism by chemotaxis inhibitor protein of *S. aureus* (CHIPS) [104]. The redundant effects of these staphylococcal molecules almost completely inactivate the complement system and facilitate immune evasion.

Professional phagocytes are the most effective cells in battling *S. aureus*. As such, *S. aureus* has evolved multiple mechanisms to avoid them by regulating their recruitment and function. For example, capsule-producing *S. aureus* are more resistant to phagocytosis and killing [105,106], induce less pro-inflammatory cytokine production [107], and are associated with worse infections [108]. Moreover, staphylococcal protein A, the most abundant protein in the staphylococcal cell wall, binds Fc receptors on the surface of phagocytes and prevents antibody-mediated phagocytosis [109]. If engulfed by phagocytic cells, *S. aureus* can resist the oxidative burst by producing superoxide dismutase and catalase to reduce superoxide and hydrogen peroxide into non-lethal compounds [110,111]. *S. aureus* can also mask the MAMPs in its PGN layer with wall teichoic acids [112] or by acetylation of the PGN backbone that resist lysozyme cleaving [113], avoiding detection by intracellular PRRs [113]. Moreover, as described above, mechanisms that prevent phagocytosis or phagosomal processing of *S. aureus* may also contribute to this immune evasion strategy by limiting the pro-inflammatory response while maintaining the IL-10 induction [22]. In fact, IL-10 itself may contribute to the modulation of phagolysosome formation and function, as seen during *Mycobacterium tuberculosis* infection [114,115]. Altogether, these evasion strategies limit the recognition and clearance of *S. aureus* by professional phagocytes*.*

More pertinent to this review is the fact that *S. aureus* can evade its host by modulating the immune response it triggers. The staphylococcal adenosine synthase A (AdsA) is a PGN-anchored protein that converts adenosine phosphates (e.g., AMP, ADP, and ATP) to adenosine, a potent anti-inflammatory molecule. *S. aureus* strains deficient in AdsA are more susceptible to neutrophil killing and cause less severe disease in mouse models of staphylococcal infections [116]. Adenosine suppresses neutrophil degranulation and extracellular trap formation [117], decreases IL-12 production, and impairs T cell stimulation by DCs [118,119]. It also directly acts on T cells to promote the differentiation into type 1 regulatory T cells (Tr1), and to enhance their IL-10 production [120]. Collectively, the anti-inflammatory effects of adenosine tailor the host adaptive immune response away from a T_H_1/T_H_17 clearance response and towards an immunomodulatory phenotype that promotes microbial survival and colonization. Therefore, AdsA is an important staphylococcal virulence factors that promotes evasion by modulating the host immune response.

In addition to the mechanisms mentioned above, *S. aureus* can also alter the response of neutrophils to promote its survival upon phagocytosis. Although neutrophils are essential for the clearance of *S. aureus*, their recruitment to the site of infection is associated with enhanced bacterial burden and pathogenicity [24]. This implies *S. aureus* has developed mechanisms to evade neutrophil function. One mechanism involves *S. aureus* upregulating the “don’t eat me” signal CD47 on neutrophils to prevent their uptake by responding macrophages [23]. This prolonged neutrophil survival enables *S. aureus* to trigger a specific type of neutrophil cell death program that releases viable *S. aureus* into the extracellular environment [23]. Thus, in certain circumstances, a strong T_H_17 response that recruits many neutrophils may end up benefiting the microbe more than the host.

The robust IL-10 response that *S. aureus* induces can also promote immune evasion through modulation of the host response in a site-dependent manner [10]. Similar to adenosine, IL-10 can down-regulate the expression of MHC class II [121] and co-stimulatory molecules [122], and can inhibit pro-inflammatory cytokine production [123] and T_H_1 responses [124]. The immune evasion effect of IL-10 is best seen in patients with staphylococcal bacteremia, where elevated IL-10 levels are associated with mortality [57]. This robust modulation of the host immune response, by suppressing macrophage and neutrophil migration and function and by down-regulating the adaptive T_H_1/T_H_17 responses, allows *S. aureus* to spread and cause severe invasive infections. A similar observation has been described for *Bordetella* [125] and may be applicable to other microbes since an IL-10 response has been linked to other PRRs in addition to TLR2.

## 7. Clinical Implications and Future Prospects

Current treatments for staphylococcal infections rely heavily on antibiotics, which will select for antibiotic-resistance strains (e.g., MRSA) [126]. Despite significant efforts, no staphylococcal vaccine has shown clinical protective efficacy [32,127,128]. Therefore, discovery of alternative anti-staphylococcal treatments is a high priority. The understanding of immunomodulation by *S. aureus* may facilitate the development of new methods to prevent and treat staphylococcal infections.

The observation of recurrent infections by *S. aureus* in a given individual suggests that the initial infection does not generate host protective immunity to subsequent infections [1]. The reason why it is so difficult to develop effective immunity against *S. aureus* can be partially explained by immunomodulation. For example, SpA binds to IgG antibodies and acts as a B cell SAg, limiting host responses to other staphylococcal virulence factors [129]. In addition, IL-10-mediated immunomodulation may also participate in the failure of *S. aureus* vaccination by limiting T cell activation and help. Therefore, purification of a low IL-10-inducing staphylococcal antigen can be considered as a strategy for vaccine development.

As discussed above, IL-10 plays an important role in *S. aureus*-induced immunomodulation, down-regulating SAg-induced T cell activation, and preventing TSS. Thus, IL-10 is a potential therapeutic agent in inflammatory diseases. Indeed, administration of recombinant IL-10 is protective in a mouse model of TSS [130,131]. However, its application in humans is limited by its low bioavailability and by its toxicity [132].

The pro-inflammatory and immunomodulatory properties of *S. aureus* can be uncoupled and are mediated by different signaling pathways [22]. One would expect that shutting down one pathway and enhancing the other using chemical inhibitors could be a treatment for staphylococcal infection. However, these signaling cascades, *i.e*., PI3K/Akt/mTOR and MAPKs, are usually involved in many aspects of cellular function. Therefore, direct manipulation of these signaling cascades is likely to cause numerous side effects. For example, pan-class I PI3K inhibitors, used to treat rheumatoid arthritis (RA), have been reported to cause hyperglycemia, gastrointestinal symptoms, and psychiatric side effects [133]. Similarly, the PI3K inhibitors used for cancer treatment also have adverse effects with a high risk of microbial infections [134,135]. MAPK p38 plays a pivotal role in pro-inflammatory responses to infections, and this provides the rationale for it being a therapeutic target. Despite the high efficacy in treating autoimmune disorders in mouse models [136], the results from clinical trials with these compounds are disappointing, as they lack long-term efficacy [137,138,139,140].

Staphylococcal cell wall-embedded TLR2 ligands are predominantly responsible for *S. aureus*-induced immunomodulation. Compared with heat-killed *S. aureus*, clindamycin-treated *S. aureus* better preserves the anti-inflammatory properties, in terms of IL-10-inducing capacity [22]. This illustrates the importance of an intact cell wall for the immunomodulation capacity of *S. aureus*. In line with this observation, clinical data show that treating TSS with bacteriostatic antibiotics (e.g., clindamycin) is more effective and has better clinical outcomes than bactericidal antibiotics [89,141].

The uncoupling of the immunomodulatory properties from the pro-inflammatory properties of *S. aureus* opens up the search for anti-inflammatory ligands in the cell wall. The study on the uncoupling of responses to *S. aureus* suggests that the staphylococcal cell wall contains qualitatively different TLR2 ligands with distinct IL-10-inducing capacities. Although the anti-inflammatory TLR2 ligands are yet to be identified, it is very unlikely that a single ligand will solely induce IL-10 without inducing pro-inflammatory cytokines. However, identification of a predominant IL-10-inducing TLR2 ligand may provide a template for novel immunomodulatory drug development.

## 8. Conclusions

*S. aureus* is very effective at immunomodulating innate and adaptive immune responses, to promote either disease tolerance or immune evasion. The type of immune response to *S. aureus* that will develop is determined by various factors, such as site of infection, bacterial virulence and host genetics, all together translating into distinct clinical outcomes. A better understanding of the mechanisms of immunomodulation leading to disease tolerance and commensalism will provide further insights into staphylococcal diseases and the development of novel clinical treatments for these conditions.
